# Fast and Guaranteed Safe Controller Synthesis for Nonlinear Vehicle Models

**DOI:** 10.1007/978-3-030-53288-8_31

**Published:** 2020-06-13

**Authors:** Chuchu Fan, Kristina Miller, Sayan Mitra

**Affiliations:** 8 Microsoft Research Lab, Redmond, WA USA; 9 University of Southern California, Los Angeles, CA USA; 10 Department of Computing and Mathematical Sciences, California Institute of Technology, Pasadena, USA; 11 Department of Electrical and Computer Engineering, University of Illinois at Urbana-Champaign, Champaign, USA

## Abstract

We address the problem of synthesizing a controller for nonlinear systems with reach-avoid requirements. Our controller consists of a reference controller and a tracking controller which drives the actual trajectory to follow the reference trajectory. We identify a type of reference trajectory such that the tracking error between the actual trajectory of the closed-loop system and the reference trajectory can be bounded. Moreover, such a bound on the tracking error is independent of the reference trajectory. Using such bounds on the tracking error, we propose a method that can find a reference trajectory by solving a satisfiability problem over linear constraints. Our overall algorithm guarantees that the resulting controller can make sure every trajectory from the initial set of the system satisfies the given reach-avoid requirement. We also implement our technique in a tool FACTEST. We show that FACTEST can find controllers for four vehicle models (3–6 dimensional state space and 2–4 dimensional input space) across eight scenarios (with up to 22 obstacles), all with running time at the sub-second range.

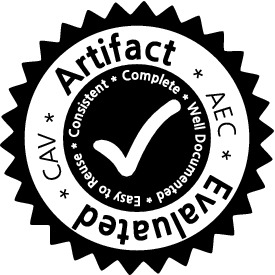



## Introduction

Design automation and safety of autonomous systems is an important research area. Controller synthesis aims to provide correct-by-construction controllers that can guarantee that the system under control meets certain requirements. Controller synthesis is a type of program synthesis problem. The synthesized program or *controller* *g* has to meet the given requirement *R*, when it is run in (closed-loop) composition with a given physical process or *plant* $\mathcal{A}$. Therefore, a synthesis algorithm has to account for the combined behavior of *g* and $\mathcal{A}$.

Methods for designing controllers for asymptotic requirements like stability, robustness, and tracking, predate the algorithmic synthesis approaches for programs [3, 16, 30]. However, these classic control design methods normally do not provide formal guarantees in terms of handling bounded-horizon requirements like safety. Typical controller programs are small, well-structured, and at core, have a succinct logic (“bang-bang” control) or mathematical operations (PID control). This might suggest that controllers could be an attractive target for algorithmic synthesis for safety, temporal logic (TL), and bounded time requirements [1, 9, 18, 34, 38].

On the other hand, *motion planning (MP),* which is an instance of the controller synthesis for robots is notoriously difficult (see [21] Chapter 6.5). A typical MP requirement is to make a robot $\mathcal{A}$ track certain waypoints while meeting some constraints. A popular paradigm in MP, called sampling-based MP, gives practical, *fully automatic,* randomized, solutions to hard problem instances by only considering the geometry of the vehicle and the free space [14, 15, 20, 21]. However, they do not ensure that the dynamic behavior of the vehicle will actually follow the planed path without running into obstacles. Ergo, MP continues to be a central problem in robotics^1^.

In this paper, we aim to achieve faster control synthesis with guarantees by exploiting a separation of concerns that exists in the problem: (A) how to drive a vehicle/plant to a *given waypoint*? and (B) Which *waypoints* to choose for achieving the ultimate goal? (A) can be solved using powerful control theoretic techniques—if not completely automatically, but at least in a principled fashion, with guarantees, for a broad class of $\mathcal{A}$’s. Given a solution for (A), we solve (B) algorithmically. A contribution of the paper is to identify characteristics of a solution of (A) that make solutions of (B) effective. Consider nonlinear control systems  and reach-avoid requirements defined by a goal set *G* that the trajectories should reach, and obstacles $\mathbf{{O}}$ the trajectories should avoid. The above separation leads to a two step process: (A) Find a state feedback tracking controller $g_\textsf {trk}$ that drives the actual trajectory of the closed-loop system $\xi _g$ to follow a reference trajectory $\xi _\textsf {ref}$. (B) Design a reference controller $g_\textsf {ref}$, which consists of a reference trajectory $\xi _\textsf {ref}$ and a reference input $u_\textsf {ref}$. The distance between $\xi _g$ and $\xi _\textsf {ref}$ is called the tracking error *e*. If we can somehow know beforehand the value of *e* without knowing $\xi _\textsf {ref}$, we can use such error to bloat $\mathbf{{O}}$ and shrink *G*, and then synthesize $\xi _\textsf {ref}$ such that it is *e* away from the obstacles (inside the goal set). For linear systems, this was the approach used in [7], but for nonlinear systems, the tracking error *e* will generally change with $\xi _\textsf {ref}$, and the two steps get entangled.

For a general class of nonlinear vehicles (such as cars, drones, and underwater vehicles), the tracking controller $g_\textsf {trk}$ is always designed to minimize the tracking error. The convergence of the error can be proved by a Lyapunov function for certain types of $\xi _\textsf {ref}$. We show how, under reasonable assumptions, we can use Lyapunov functions to bound the value of the tracking error *even when the waypoints changes* (Lemma 2). This error bound is independent of $\xi _\textsf {ref}$ so long as $\xi _\textsf {ref}$ satisfies the assumptions. For step (B) we introduce a SAT-based trajectory planning methods to find such $\xi _\textsf {ref}$ and $u_\textsf {ref}$ by solving a satisfiability (SAT) problem over quantifier free linear real arithmetic (Theorem 1). Moreover, the number of constraints in the SMT problem scales linearly to the increase of number of obstacles (and not with the vehicle model). Thus, our methods can scale to complex requirements and high dimensional systems.

Putting it all together, our final synthesis algorithm (Algorithm 2) guarantees that any trajectory following the synthesized reference trajectory will satisfy the reach-avoid requirements. The resulting tool FACTEST is tested with four nonlinear vehicle models and on eight different scenarios, taken from MP literature, which cover a wide range of 2D and 3D environments. Experiment results show that our tool scales very well: it can find the small covers $\{ \Theta _j \}_j$ and the corresponding reference trajectories and control inputs satisfying the reach-avoid requirements most often in less than a second, even with up to 22 obstacles. We have also compared our SAT-based trajectory planner to a standard RRT planner, and the results show that our SAT-based method resoundingly outperforms RRT. To summarize, our main contributions are:

1. A method (Algorithm 2) for controller synthesis separating tracking controller $g_\textsf {trk}$ and search for reference controller $g_\textsf {ref}$.
2. Sufficient conditions for tracking controller error performance that makes the decomposition work (Lemma 2 and Lemma 3).
3. An SMT-based effective method for synthesizing reference controller $g_\textsf {ref}$.
4. The FACTEST implementation of the above and its evaluation showing very encouraging results in terms of finding controllers that make any trajectories of the closed-loop system satisfy reach-avoid requirements (Sect. 6).

**Related Works.** *Model Predictive Control (MPC).* MPC [4, 25, 45, 49] has to solve a constrained, discrete-time, optimal control problem. MPC for controller synthesis typically requires model reduction for casting the optimization problem as an LP [4], QP [2, 36], MILP [33, 34, 45]. However, when the plant model is nonlinear [8, 22], it may be hard to balance speed and complex requirements as the optimization problem become nonconvex and nonlinear.

*Discrete Abstractions.* Discrete, finite-state, abstraction of the control system is computed, and then a discrete controller is synthesized by solving a two-player game [10, 17, 24, 42, 47]. CoSyMA [28], Pessoa [37], LTLMop [18, 46], Tulip [9, 48], and SCOTS [38] are based on these approaches. The discretization step often leads to a severe state space explosion for higher dimensional models.

*Safe Motion Planning.* The idea of bounding the tracking error through pre-computation has been used in several techniques: FastTrack [11] uses Hamilton-Jacobi reachability analysis to produce a “safety bubble” around planed paths. Reachability based trajectory design for dynamical environments (RTD) [44] computes an offline forward reachable sets to guarantee that the robot is not-at-fault in any collision. In [40], a technique based on convex optimization is used to compute tracking error bounds. Another technique [23, 43] uses motion primitives expanded by safety funnels, which defines similar ideas of safety tubes.

*Sampling Based Planning.* Probabilistic Road Maps (PRM) [15], Rapidly-exploring Random Trees (RRT) [19], and fast marching tree (FMT) [12] are widely used in actual robotic platforms. They can generate feasible trajectories through known or partially known environments. Compared with the deterministic guarantees provided by our proposed method, these methods come with stochastic guarantees. Also, they are not designed to be robust to model uncertainty or disturbances. MoveIT [5] is a tool designed to implement and benchmark various motion planners on robots. The motion planners in MoveIT are from the open motion planning library (OMPL) [41], which implements motion planners abstractly.

*Controlled Lyapunov Function (CLF).* CLF have been used to guarantee that the overall closed-loop controlled system satisfies a reach-while-stay specification [35]. Instead of asking for a CLF for the overall closed-loop system, our method only needs a Lyapunov function for the tracking error, which is a weaker local requirement. CLF is often a difficult requirement to meet for nonlinear vehicle models.

## Preliminaries and Problem Statement

Let us denote real numbers by ${\mathbb R}$, non-negative real numbers by ${{\mathbb R}_{\ge 0} }$, and natural numbers by ${\mathbb N}$. The *n*-dimensional *Euclidean space* is ${\mathbb R}^n$. For a vector $x \in {\mathbb R}^n$, $x^{(i)}$ is the $i^{th}$ entry of *x* and $\Vert x \Vert _2$ is the 2-norm of *x*. For any matrix $A \in {\mathbb R}^{n \times m}$, $A^{\intercal }$ is its *transpose*; ${A}^{(i)}$ is the $i^{th}$ row of *A*. Given a $r \ge 0$, an *r*-*ball* around $x \in {\mathbb R}^n$ is defined as $B_r(x) = \{x' \in {\mathbb R}^n \ | \ ||x' - x ||_2 \le r \}$. We call *r* the radius of the ball. Given a matrix $H \in {\mathbb R}^{r \times n}$ and a vector $b \in {\mathbb R}^r$, an (*H*, *b*)-*polytope* is denoted by $Poly(H,b) = \{x\in {\mathbb R}^n \ | \ Hx \le b \}$. Each row of the inequality $H^{(i)} x \le b^{(i)}$ defines a *halfspace*. We also call $H^{(i)} x = b^{(i)}$ the *surface* of the polytope. Let $\mathsf {dP}(H) = r$ denotes the number of rows in *H*. Given a set $S \subseteq {\mathbb R}^n$, the radius of *S* is defined as $\sup _{x,y\in S}\Vert x-y\Vert _2/2$.

*State Space and Workspace.* The state space of control systems will be a subspace $\mathcal{X}\subseteq {\mathbb R}^n$. The *workspace* is a subspace $\mathcal{W}\subseteq {\mathbb R}^d$, for $d \in \{2,3\}$, which is the physical space in which the robots have to avoid obstacles and reach goals. Given a state vector $x \in \mathcal{X}$, its projection to $\mathcal{W}$ is denoted by $x \downarrow p$. That is, $x \downarrow p = [p_x, p_y]^\intercal \in {\mathbb R}^2$ for ground vehicles on the plane and $x \downarrow p = [p_x, p_y, p_z]^\intercal \in {\mathbb R}^3$ for aerial and underwater vehicles. When *x* is clear from context we will write $x \downarrow p$ as simply *p*. The vector *x* may include other variables like velocity, heading, pitch, etc., but *p* only has the position in Cartesian coordinates. We assume that the goal set $G:= Poly(H_{\scriptscriptstyle {G}}, b_{\scriptscriptstyle {G}})$ and the unsafe set $\mathbf{{O}}$ (obstacles) are specified by polytopes in $\mathcal{W}$; $\mathbf{{O}}= \cup O_i$, where $O_i := Poly(H_{{\scriptscriptstyle {O}},i}, b_{{\scriptscriptstyle {O}},i})$ for each obstacle *i*.

**Fig. 1.**
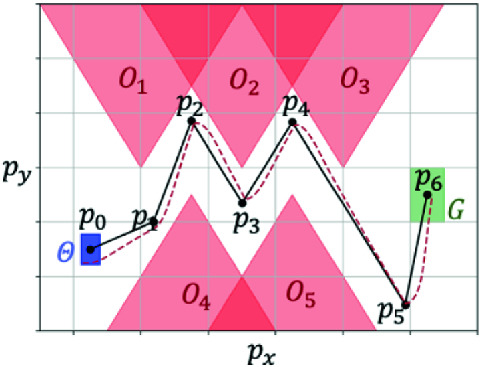
Zigzag scenario for a controller synthesis problem. The initial set is blue, the goal set is green, and the unsafe sets are red. A valid reference trajectory is shown in black and a feasible trajectory is shown in purple. (Color figure online)

*Trajectories and Reach-Avoid Requirements.* A *trajectory* $\xi$ over $\mathcal{X}$ of duration *T* is a function $\xi : [0, T] \rightarrow \mathcal{X}$, that maps each time *t* in the time *domain* [0, *T*] to a point $\xi (t) \in \mathcal{X}$. The *time bound or duration* of $\xi$ is denoted by $\xi .\textsf {ltime}= T$. The projection of a trajectory $\xi : [0, T] \rightarrow \mathcal{X}$ to $\mathcal{W}$ is written as $\xi \downarrow p : [0, T] \rightarrow \mathcal{W}$ and defined as $(\xi \downarrow p)(t) = \xi (t) \downarrow p$. We say that a trajectory $\xi (t)$ *satisfies a reach-avoid requirement given by unsafe set* $\mathbf{{O}}$ *and goal set* *G*if $\forall t \in [0,\xi .\textsf {ltime}], \xi (t) \downarrow p \notin \mathbf{{O}}$ and $\xi (\xi .\textsf {ltime})\downarrow p \in G$. See Fig. 1 for an example.

Given a trajectory $\xi : [0, T] \rightarrow \mathcal{X}$ and a time $t > 0$, the *time shift* of $\xi$ is a function $(\xi + t) : [t, t + T] \rightarrow \mathcal{X}$ defined as $\forall t' \in [t, t + T], (\xi + t)(t') = \xi (t' - t)$. Strictly speaking, for $t > 0, \xi + t$ is not a trajectory. The *concatenation* of two trajectories $\xi _{1} \frown \xi _{2}$ is a new trajectory in which $\xi _{1}$ is followed by $\xi _{2}$. That is, for each $t \in [0, \xi _{1}.\textsf {ltime}+ \xi _{2}.\textsf {ltime}]$, $(\xi _{1} \frown \xi _{2})(t) = \xi _{1}(t)$ when $t \le \xi _{1}.\textsf {ltime}$, and equals $\xi _{2}(t - \xi _{1}.\textsf {ltime})$ when $t > \xi _{1}.\textsf {ltime}$. Trajectories are closed under concatenation, and many trajectories can be concatenated in the same way.

### Nonlinear Control System

#### Definition 1

An (*n*, *m*)*-dimensional control system* $\mathcal{A}$ is a 4-tuple $\langle \mathcal {X}, \Theta , \mathbf {U}, f \rangle$ where (i) $\mathcal{X}\subseteq \mathbb {R}^n$ is the state space, (ii) $\Theta \subseteq \mathcal{X}$ is the initial set, (iii) $\mathbf {U}\subseteq \mathbb {R}^m$ is the input space, and (iv) $f: \mathcal{X}\times \mathbf {U}\rightarrow \mathcal{X}$ is the *dynamic function* that is Lipschitz continuous with respect to the first argument.

A control system with no inputs ($m = 0$) is called a *closed* system.

Let us fix a time duration $T > 0$. An *input trajectory* $u : [0,T] \rightarrow \mathbf {U}$, is a continuous trajectory over the input space $\mathbf {U}$. We denote the set of all possible input trajectories to be $\mathcal{U}$. Given an input signal $u \in \mathcal{U}$ and an initial state $x_0 \in \Theta$, a *solution* of $\mathcal {A}$ is a continuous trajectory $\xi _u : [0,T] \rightarrow \mathcal{X}$ that satisfies (i) $\xi _u(0) = x_0$ and (ii) for any $t \in [0,T]$, the time derivative of $\xi _u$ at *t* satisfies the differential equation:1For any $x_0 \in \Theta , u\in \mathcal{U}$, $\xi _u$ is a state trajectory and we call such a pair $(\xi _u, u)$ a state-input trajectory pair.

A *reference state trajectory* (or *reference trajectory* for brevity) is a trajectory over $\mathcal{X}$ that the control system tries to follow. We denote reference trajectories by $\xi _\textsf {ref}$. Similarly, a *reference input trajectory* (or *reference input*) is a trajectory over $\mathbf {U}$ and we denote them as $u_\textsf {ref}$. Note these $\xi _\textsf {ref}$ and $u_\textsf {ref}$ are not necessarily solutions of (1). Figure 1 shows reference and actual solution trajectories.

We call a reference trajectory $\xi _\textsf {ref}$ and a reference input $u_\textsf {ref}$ together as a reference controller $g_\textsf {ref}$. Given $g_\textsf {ref}$, a *tracking controller* $g_{{{\textsf {\textit{trk}}}}}$ is a function that is used to compute the inputs for $\mathcal{A}$ so that in the resulting closed system, the state trajectories try to follow $\xi _\textsf {ref}$.

#### Definition 2

Given an (*n*, *m*)-dynamical system $\mathcal{A}$, a reference trajectory $\xi _\textsf {ref}$, and a reference input $u_\textsf {ref}$, a *tracking controller* for the triple $\langle \mathcal{A}, \xi _\textsf {ref}, u_\textsf {ref}\rangle$ is a (state feedback) function $g_\textsf {trk}: \mathcal{X}\times \mathcal{X}\times \mathbf {U}\rightarrow \mathbf {U}$.

At any time *t*, the tracking controller $g_\textsf {trk}$ takes in a current state of the system *x*, a reference trajectory state $\xi _\textsf {ref}(t)$, and a reference input $u_\textsf {ref}(t)$, and gives an input $g_\textsf {trk}(x,\xi _\textsf {ref}(t), u_\textsf {ref}(t)) \in \mathbf {U}$ for $\mathcal{A}$. The controller *g* for $\mathcal{A}$ is determined by both the reference controller $g_\textsf {ref}$ and the tracking controller $g_\textsf {trk}$. The resulting trajectory $\xi _g$ of the closed control system ($\mathcal{A}$ closed with $g_\textsf {ref}$ and $g_\textsf {trk}$) satisfies:2where *D* is the set of points in time where the second or third argument of $g_\textsf {trk}$ is discontinuous^2^.

### Controller Synthesis Problem

#### Definition 3

Given a (*n*, *m*)-dimensional nonlinear system $\mathcal{A}= \langle \mathcal {X}, \Theta , \mathbf {U}, f \rangle$, its workspace $\mathcal{W}$, goal set $G \subseteq \mathcal{W}$ and the unsafe set $\mathbf{{O}}\subseteq \mathcal{W}$, we are required to find (a) a tracking controller $g_\textsf {trk}$, (b) a partition $\{\Theta _j\}_j$ of $\Theta$, and (c) for each partition $\Theta _j$, a reference controller $g_{j,\textsf {ref}}$, which consists of a state trajectory $\xi _{j,\textsf {ref}}$ and an input trajectory $u_{j,\textsf {ref}}$, such that $\forall x_0 \in \Theta _j$, the unique trajectory $\xi _g$ of the closed system as in Eq. (2) starting from $x_0$ reaches *G* and avoids $\mathbf{{O}}$.

Again, $\xi _{j,\textsf {ref}}$ and $u_{j,\textsf {ref}}$ in $g_{j, \textsf {ref}}$ are not required to be a state-input pair, but, for each initial state $x_0 \in \Theta _j$, the closed loop trajectory $\xi _g$ following $\xi _\textsf {ref}$ *is* a valid state trajectory with corresponding input *u* generated by $g_\textsf {trk}$ and $g_{j,\textsf {ref}}$. In this paper, we will decompose the controller synthesis problem: Part (a) will be delivered by design engineers with knowledge of vehicle dynamics, and parts (b) and (c) will be automatically synthesized by our algorithm. The latter being the main contribution of the paper.

#### Example 1

Consider a ground vehicle moving on a 2D workspace $\mathcal{W}\subseteq {\mathbb R}^2$ as shown in Fig. 1.

This scenario is called Zigzag and it is adopted from [32]. The red polytopes are obstacles. The blue and green polytopes are the initial set $\Theta$ and the goal set *G*. There are also obstacles (not shown in the figure) defining the boundaries of the entire workspace. The black line is a projection of a reference trajectory to the workspace: $\xi _\textsf {ref}(t) \downarrow p$. This would not be a feasible state trajectory for a ground vehicle that cannot make sharp turns. The purple dashed curve is a real feasible state trajectory of the system starting from $\Theta$ with a tracking controller $g_\textsf {trk}$, where $g_\textsf {trk}$ will be introduced in Example 2.

Consider the standard nonlinear bicycle model of a car [31]. The control system has 3 state variables: the position $p_x, p_y$, and the heading direction $\theta$. Its motion is controlled by two inputs: linear velocity *v* and rotational velocity $\omega$. The car’s dynamics are given by:3


## Constructing Reference Trajectories from Waypoints

If $\xi _\textsf {ref}(t) \downarrow p$ is a PWL (PWL) curve in the workspace $\mathcal{W}$, we call $\xi _\textsf {ref}(t)$ a PWL reference trajectory. In $\mathcal{W}$, a PWL curve can be determined by the endpoints of each line segment. We call such endpoints the *waypoints* of the PWL reference trajectory. In Fig. 1, the black points $p_0,\cdots ,p_6$ are waypoints of $p(t) = \xi _\textsf {ref}(t) \downarrow p$.

Consider any vehicle on the plane^3^ with state variables $p_x, p_y, \theta , v$ (*x*-position, *y*-position, heading direction, linear velocity) and input variables $a, \omega$ (acceleration and angular velocity). Once the waypoints $\{p_i\}_{i=0}^{k}$ are fixed, and if we enforce constant speed $\bar{v}$ (i.e., $\xi _\textsf {ref}(t) \downarrow v = \bar{v}$ for all $t \in [0,\xi _\textsf {ref}.\textsf {ltime}]$), then $\xi _\textsf {ref}(t)$ can be uniquely defined by $\{p_i\}_{i=0}^{k}$ and $\bar{v}$ using Algorithm 1. The semantics of $\xi _\textsf {ref}$ and $u_\textsf {ref}$ returned by  is that the reference trajectory requires the vehicle to move at a constant speed $\bar{v}$ along the lines connecting the waypoints $\{p_i\}_{i=0}^{k}$. In Example 1, $\xi _\textsf {ref}(t), u_\textsf {ref}(t)$ can also be constructed using  moving *v* to input variables and dropping *a*.

We notice that if $k = 1$, $\xi _\textsf {ref}(t), u_\textsf {ref}(t)$ returned by Algorithm 1 is a valid state-input trajectory pair. However, if $k > 1$, $\xi _\textsf {ref}(t), u_\textsf {ref}(t)$ returned by Algorithm 1 is usually not a valid state-input trajectory pair. This is because $\theta _\textsf {ref}{(t)}$ is discontinuous at the waypoints and no bounded inputs $u_\textsf {ref}(t)$ can drive the vehicle to achieve such $\theta _\textsf {ref}(t)$. Therefore, when $k>1$, $\xi _\textsf {ref}(t)$ is a PWL reference trajectory with no $u_\textsf {ref}(t)$ such that $\xi _\textsf {ref}, u_\textsf {ref}$ are solutions of (1).
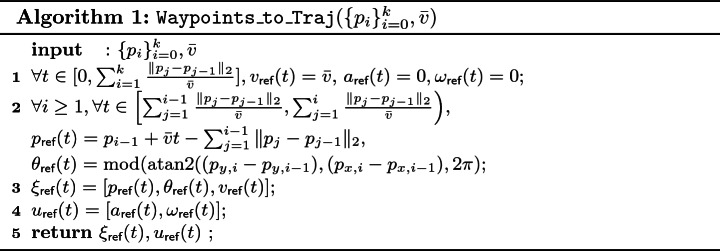


### Proposition 1

Given a sequence of waypoints $\{p_i\}_{i=0}^{k}$ and a constant speed $\bar{v}$, $\xi _\textsf {ref}(t), u_\textsf {ref}(t)$ produced by  satisfy:

- $p_\textsf {ref}(t) = \xi _\textsf {ref}(t)\downarrow p$ is a piece-wise continuous function connecting $\{p_i\}_{i=0}^{k}$.
- At time $t_i = \sum _{j=1}^{i} \Vert p_j-p_{j-1} \Vert _2/{\bar{v}}$, $p_\textsf {ref}(t_i) = p_i$. We call $\{t_i\})_{i=1}^{k}$ the concatenation time.
- $\xi _\textsf {ref}(t) = \xi _{\textsf {ref},1}(t) \frown \cdots \frown \xi _{\textsf {ref},k}(t)$ and $u_\textsf {ref}(t) = u_{\textsf {ref},1}(t) \frown \cdots \frown u_{\textsf {ref},k}(t)$, where $(\xi _{\textsf {ref},i}, u_{\textsf {ref},i})$ are state-input trajectory pairs returned by the function .

**Outline of Synthesis Approach.** In this Section, we present an Algorithm  for constructing reference trajectories from arbitrary sequence of waypoints. In Sect. 4, we precisely characterize the type of vehicle tracking controller our method requires from designers. On our tool’s webpage [27], we show with several extra examples that indeed developing such controllers is non-trivial, far from automatic, yet bread and butter of control engineers. In Sect. 5, we present the main synthesis algorithm, which uses the tracking error bounds from the previous section, to construct waypoints, for each initial state, which when passed through  provide the solutions to the synthesis problem.

## Bounding the Error of a Tracking Controller

### Tracking Error and Lyapunov Functions

Given a reference controller $g_\textsf {ref}$, a tracking controller $g_\textsf {trk}$, and an initial state $x_0 \in \Theta$, the resulting trajectory $\xi _g$ of the closed control system ($\mathcal{A}$ closed with $g_\textsf {ref}$ and $g_\textsf {trk}$) is a state trajectory that starts from $x_0$ and follows the ODE (2). In this setting, we define the *tracking error* at time *t* to be a continuous function:

$$\begin{aligned} e: \mathcal{X}\times \mathcal{X}\rightarrow {\mathbb R}^n. \end{aligned}$$

When $\xi _g(t)$ and $\xi _\textsf {ref}(t)$ are fixed, we also write $e(t) = e(\xi _g(t),\xi _\textsf {ref}(t))$ which makes it a function of time. One thing to remark here is that if $\xi _\textsf {ref}(t)$ is discontinuous, then *e*(*t*) is also discontinuous. In this case, the derivative of *e*(*t*) cannot be defined at the points of discontinuity. To start with, let us assume that $g_\textsf {ref}= (\xi _\textsf {ref},u_\textsf {ref})$ is a valid state-input pair so $\xi _\textsf {ref}$ is a continuous state trajectory. Later we will see that the analysis can be extended to cases when $\xi _\textsf {ref}$ is discontinuous but a concatenation of continuous state trajectories.

When $(\xi _\textsf {ref},u_\textsf {ref})$ is a valid state-input pair and *e*(*t*) satisfy an differential equation $\frac{d}{dt}{e(t)} = f_e(e(t))$, we use Lyapunov functions, which is a classic technique for proving stability of an equilibrium of an ODE, to bound the tracking error *e*(*t*). The Lie derivative $\frac{\partial V}{\partial e} f_e(e)$ below captures the rate of change of the function *V* along the trajectories of *e*(*t*).

#### Definition 4

**(Lyapunov functions** [16]**).** Fix a state-input reference trajectory pair $(\xi _\textsf {ref},u_\textsf {ref})$, assume that the dynamics of the tracking error *e* for a closed control system $\mathcal{A}$ with $g_\textsf {ref}$ and $g_\textsf {trk}$ can be rewritten as $\frac{d}{dt}e(t) = f_e(e(t))$, where $f_e(0) = 0$. A continuously differentiable function $V: {\mathbb R}^n \rightarrow {\mathbb R}$ satisfying (i) $V(0) = 0,$ (ii) $\forall e \in {\mathbb R}^n, V(e) \ge 0,$ and (iii) $\forall e \in {\mathbb R}^n, \frac{\partial V}{\partial e} f_e(e) \le 0$, is called a Lyapunov function for the tracking error.

#### Example 2

For the car of Example 1, with a continuous reference trajectory $\xi _\textsf {ref}(t) = [x_\textsf {ref}(t), y_\textsf {ref}(t), \theta _\textsf {ref}(t)]^\intercal$, we define the tracking error in a coordinate frame fixed to the car [13]:4With the reference controller function *g* defined as:5it has been shown in [13] when $k_1,k_2,k_3 > 0$, , and ,6is a Lyapunov function with negative semi-definite time derivative .

### Bounding Tracking Error Using Lyapunov Functions: Part 1

Consider a given closed control system, $\mathcal{A}$ with $g_\textsf {ref}$ and $g_\textsf {trk}$, in this section, we will derive upper bounds on the tracking error *e*. Later in Sect. 5, we will develop techniques that take the tracking error into consideration for computing reference trajectories $\xi _\textsf {ref}$.

To begin with, we consider state-input reference trajectory pairs $(\xi _\textsf {ref}, u_\textsf {ref})$ where $u_\textsf {ref}$ is continuous, and therefore, $\xi _\textsf {ref}$ and $\xi _g$ are differentiable. Let us assume that the tracking error dynamics () has a Lyapunov function *V*(*e*(*t*)). The following is a standard result that follows from the theory of Lyapunov functions for dynamical systems.

#### Lemma 1

Consider any state-input trajectory pair $(\xi _\textsf {ref}, u_\textsf {ref})$, an initial state $x_0$, the corresponding trajectory $\xi _g$ of the closed control system, and a constant $\ell > 0$. If the tracking error *e*(*t*) has a Lyapunov function *V*, and if initially $V(e(0)) \le \ell ,$ then for any $t \in [0, \xi _\textsf {ref}.\textsf {ltime}]$, $V(e(t)) \le \ell$.

This lemma is proved by showing that . The last inequality holds since  for any $\tau \in [0,t]$ according the definition of Lyapunov functions (Definition 4).

Lemma 1 says that if we can bound $V(e(0)) = V(e(x_0, \xi _\textsf {ref}(0)))$, we can bound $V(e(\xi _g(t), \xi _\textsf {ref}(t)))$ at any time *t* within the domain of the trajectories, regardless of the value of $\xi _\textsf {ref}(t)$. This could decouple the problem of designing the tracking controller $g_\textsf {trk}$ and synthesizing the reference controller $g_\textsf {ref}$ as a state-input trajectory pair $(\xi _\textsf {ref}, u_\textsf {ref})$.

#### Example 3

Given two waypoints $p_0, p_1$ for the car in Example 1, take the returned value of , move $v_\textsf {ref}$ to $u_\textsf {ref}$ and drop $a_\textsf {ref}$. Then, the resulting $(\xi _\textsf {ref},u_\textsf {ref})$ is a continuous and differentiable state-input reference trajectory pair. Moreover, if the robot is controlled by the tracking controller as in Eq. (5),  is a Lyapunov function for the corresponding tracking error $e(t) = [e_x(t), e_y(t), e_\theta (t)]^\intercal$.

From Eq. (4), it is easy to check that $e_x^2(t) + e_y(t)^2 = (x_\textsf {ref}(t) -p_x(t))^2 + (y_\textsf {ref}(t) - p_y(t)) ^2$ for any time *t*. Assume that initially the position of the vehicle satisfies $[p_x(0), p_y(0)]^\intercal \in B_{\ell }([x_\textsf {ref}(0), y_\textsf {ref}(0)]^\intercal )$. We check that .

From Lemma 1, we know that $\forall t \in [0,\xi _\textsf {ref}.\textsf {ltime}]$, . Then we have $(x_\textsf {ref}(t) -p_x(t))^2 + (y_\textsf {ref}(t) - p_y(t)) ^2 = (e_x(t)^2 + e_y(t)^2) \le \ell ^2 + \frac{4}{k_2}$. That is, the position of the robot at time *t* satisfies $[p_x(t), p_y(t)]^\intercal \in B_{\sqrt{\ell ^2+ \frac{4}{k_2}}}([x_\textsf {ref}(t), y_\textsf {ref}(t)]^\intercal )$.

### Bounding Tracking Error Using Lyapunov Functions: Part 2

Next, let us consider the case where $\xi _\textsf {ref}$ is discontinuous. Furthermore, let us assume that it is a concatenation of several continuous state trajectories $\xi _{\textsf {ref},1} \frown \cdots \frown \xi _{\textsf {ref},k}$. In this case, we call $\xi _\textsf {ref}$ a piece-wise reference trajectory. If we have a sequence of $(\xi _{\textsf {ref},i},u_{\textsf {ref},i})$, each is a valid state-input trajectory pair and the corresponding error $e_i(t)$ has a Lyapunov function $V_i(e_i(t))$, then we can use Lemma 1 to bound the error of $e_i(t)$ if we know the value of $e_i(0)$. However, the main challenge to glue these error bounds together is that *e*(*t*) would be discontinuous with respect to the entire piece-wise $\xi _\textsf {ref}(t)$.

Without loss of generality, let us assume that the Lyapunov functions $V_i(e_i(t))$ share the same format. That is, $\forall i, V_i(e_i(t)) = V(e_i(t))$. Let $t_i$ be the concatenation time points when $\xi _\textsf {ref}(t)$ (and therefore *e*(*t*)) is discontinuous. We know that $\lim _{t \rightarrow t_i^{-}}V(e(t)) \ne \lim _{t \rightarrow t_i^{+}}V(e(t))$ since $\lim _{t \rightarrow t_i^{-}}e(t) \ne \lim _{t \rightarrow t_i^{+}}e(t)$.

One insight we can get from Example 3 is that although *e*(*t*) is discontinuous at time $t_i$s, some of the variables influencing *e*(*t*) are continuous. For example, $e_x(t)$ and $e_y(t)$ in Example 3, which represent the error of the positions, are continuous since both the actual and reference positions of the vehicle are continuous. If we can further bound the term in *V*(*e*(*t*)) that corresponds to the *other* variables, we could analyze the error bound for the entire piece-wise reference trajectory. With this in sight, let us write *e*(*t*) as $[e_p(t), e_r(t)]$, where $e_p(t) = e(t)\downarrow p$ is the projection to $\mathcal{W}$ and $e_r(t)$ is the remaining components.

Let us further assume that the Lyapunov function can be written in the form of $V(e(t)) = \alpha (e_p(t)) + \beta (e_r(t))$. Indeed, on the tool’s webpage [27] we show that four commonly used vehicle models (car, robot, underwater vehicle, and hovercraft) have Lyapunov functions for the tracking error *e*(*t*) of this form. If $\beta (e_r(t))$ can be further bounded, then the tracking error for the entire trajectory can be bounded using the following lemma.

#### Lemma 2

Consider $\xi _\textsf {ref}= \xi _{\textsf {ref},1} \frown \cdots \frown \xi _{\textsf {ref},k}$, and $u_\textsf {ref}= u_{\textsf {ref},1} \frown \cdots \frown u_{\textsf {ref},k}$ as a piecewise reference and input with each $(\xi _{\textsf {ref},i},u_{\textsf {ref},i})$ being a state-input trajectory pair. Suppose (1) $V(e(t)) = \alpha (e_p(t)) + \beta (e_r(t))$ be a Lyapunov function for the tracking error *e*(*t*) of each piece $(\xi _{\textsf {ref},i},u_{\textsf {ref},i})$; (2) $e_p(t)$ is continuous and $\alpha (\cdot )$ is a continuous function; (3) $\beta (e_r(t)) \in [b_l, b_u]$, and (4) $V(e(0)) \le {\varepsilon }_0$. Then, the tracking error *e*(*t*) with respect to $\xi _\textsf {ref}$ and $u_\textsf {ref}$ can be bounded by,

$$\begin{aligned} V(e(t)) \le {\varepsilon }_{i}, \forall i \ge 1, \forall t \in [t_{i-1},t_{i}), \end{aligned}$$

where $\forall \ i >1, {\varepsilon }_i = {\varepsilon }_{i-1} - b_l + b_u$, ${\varepsilon }_1 = {\varepsilon }_0$ being the bound on the initial tracking error, and $t_i$’s are the time points of concatenation^4^.

#### Proof

We prove this by induction on *i*. When $i = 1$, we know from Lemma 1 that if the initial tracking error is bounded by *V*(*e*(0)), then for any $t \in [0,t_1), V(e(t)) \le V(e(0)) \le {\varepsilon }_0={\varepsilon }_1$, so the lemma holds.

Fix any $i \ge 1$. If $V(e(t_{i-1})) \le {\varepsilon }_{i}$, from Lemma 1 we have $\forall t \in [t_{i-1}, t_{i})$, $V(e(t)) \le {\varepsilon }_{i}$. Also, $\lim _{t \rightarrow t_i^{-}} V(e(t)) = \lim _{t \rightarrow t_i^{-}} \alpha (e_p(t)) + \beta (e_r(t)) \le {\varepsilon }_{i}$. Since $\forall e_r(t) \in {\mathbb R}^{n-d}, \beta (e_r(t)) \in [b_l, b_u]$, we have $\lim _{t \rightarrow t_i^{-}} \alpha (e_p(t)) \le {\varepsilon }_{i} - b_l,$ and $\lim _{t \rightarrow t_i^{-}} \alpha (e_p(t)) = \lim _{t \rightarrow t_i^{+}} \alpha (e_p(t))$. Therefore,

$$\begin{aligned} {\varepsilon }_{i+1} = \lim _{t \rightarrow t_i^{+}} V(e(t)) = \lim _{t \rightarrow t_i^{+}} \alpha (e_p(t)) + \beta (e_r(t)) \le {\varepsilon }_{i} -b_l + b_u. \end{aligned}$$

Another observation we have on the four vehicle models used in this paper is that not only *V*(*e*(*t*)) can be written as $\alpha (e_p(t)) + \beta (e_r(t))$ with $\beta (e_r(t))$ being bounded, but also $\alpha (e_p(t))$ can be written as $\alpha (e_p(t)) = c e_p^\intercal (t) e_p(t) = c \Vert p(t) - p_\textsf {ref}(t) \Vert _2^2$, where $c \in {\mathbb R}$ is a scalar constant; $p(t) = \xi _g(t)\downarrow p$ and $p_\textsf {ref}(t) = \xi _\textsf {ref}(t) \downarrow p$ are the actual position and reference position of the vehicle. In this case, we can further bound the position of the vehicle *p*(*t*).

#### Lemma 3

In addition to the assumptions of Lemma 2, if $\alpha (e_p(t)) = c e_p^\intercal (t) e_p(t) = c \Vert p(t) - p_\textsf {ref}(t) \Vert _2^2$, where $c \in {\mathbb R}, p(t) = \xi _g(t)\downarrow p$ and $p_\textsf {ref}(t) = \xi _\textsf {ref}(t) \downarrow p$. Then we have that at time $t \in [t_{i-1},t_{i})$,where ${\varepsilon }_i$ and $b_l$ are from Lemma 2, which implies that

$$\begin{aligned} p(t) \in B_{\ell _i}(p_\textsf {ref}(t)), with \ \ell _i = \sqrt{\frac{{\varepsilon }_i-b_l}{c}}. \end{aligned}$$

Note that Lemma 2 and 3 does not depend on the concrete values of $\xi _\textsf {ref}$ and $u_\textsf {ref}$. The lemmas hold for any piece-wise reference trajectory $\xi _\textsf {ref}$ and reference input $u_\textsf {ref}$ as long as the corresponding error *e* has a Lyapunov function (for each piece of $\xi _\textsf {ref}$ and $u_\textsf {ref}$).

**Fig. 2.**
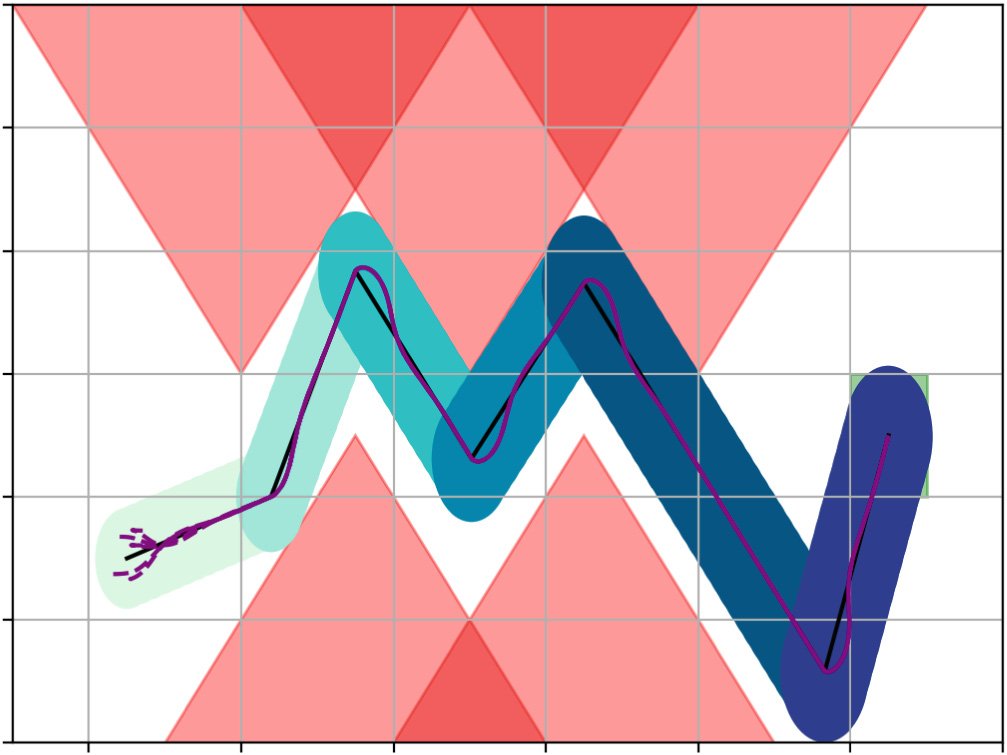
Illustration of the error bounds computed from Lemma 3. The $i^{th}$ line segment is bloated by $\sqrt{\ell ^2+ \frac{4{i}}{k_2}}$. The closed-loop system’s trajectory *p*(*t*) are purple curves and they are contained by the bloated-tube. (Color figure online)

#### Example 4

Continue Example 3.

Now let us consider the case of a sequence of waypoints $\{p_i\}_{i=0}^{k}$. Let . From Example 3, we know that $V(e(t)) = \frac{1}{2}(e_x(t)^2 + e_y(t)^2) + \frac{1-\cos (e_\theta (t))}{k_2}$ is a Lyapunov function for each segment of the piece-wise reference trajectory $\xi _\textsf {ref}(t)$. We also know that for any value of $e_\theta$, the term $\frac{1-\cos (e_\theta (t))}{k_2} \in [0,\frac{2}{k}]$. From Lemma 2, we have that for $t \in [t_{i-1}, t_i)$ where $t_i$ are the concatenation time points, we have $V(e(t)) \le V(e(0)) + \frac{2(i-1)}{k_2}$ Therefore, following Example 3, initially $V(e(0)) \le \frac{\ell ^2}{2} + \frac{2}{k_2}$. Then $\forall t \in [t_{i-1}, t_i)$, $V(e(t)) \le \frac{\ell ^2}{2} + \frac{2i}{k_2}$, and the position of the robot satisfies $[p_x(t), p_y(t)]^\intercal \in B_{\sqrt{\ell ^2+ \frac{4{i}}{k_2}}}([x_\textsf {ref}(t), y_\textsf {ref}(t)]^\intercal )$.

As seen in Fig. 2, we bloat the black reference trajectory $p_\textsf {ref}(t) = \xi _\textsf {ref}(t) \downarrow p$ by $\ell _i = \sqrt{\ell ^2+ \frac{4{i}}{k_2}}$ for the $i^{th}$ line segment, the bloated tube contains the real position trajectories (purple curves) *p*(*t*) of the closed system.

## Synthesizing the Reference Trajectories

In Sect. 4.3, we have seen that under certain conditions, the tracking error *e*(*t*) between an actual closed-loop trajectory $\xi _g(t)$ and a piece-wise reference $\xi _\textsf {ref}(t)$ can be bounded by a piece-wise constant value, which depends on the initial tracking error *e*(0) and the number of segments in $\xi _\textsf {ref}$. We have also seen an example nonlinear vehicle model with PWL $\xi _\textsf {ref}$ for which the tracking error can be bounded.

In this section, we discuss how to utilize such bound on *e*(*t*) to help find a reference controller $g_\textsf {ref}$ consisting of a reference trajectory $\xi _\textsf {ref}(t)$ and a reference input $u_\textsf {ref}(t)$ such that closed-loop trajectories $\xi _g(t)$ from a neighborhood of $\xi _\textsf {ref}(0)$ that are trying to follow $\xi _\textsf {ref}(t)$ are guaranteed to satisfy the reach-avoid requirement. The idea of finding a $g_\textsf {ref}$ follows a classic approach in robot motion planning. The intuition is that if we know at any time $t \in [0, \xi _\textsf {ref}.\textsf {ltime}]$, $\Vert \xi _g(t)\downarrow p - \xi _\textsf {ref}(t)\downarrow p \Vert _2$ will be at most $\ell$, then instead of requiring $\xi _\textsf {ref}(t)\downarrow p$ to be at least $\ell$ away from the obstacles (inside the goal region), we will bloat the obstacles (shrink the goal set) by $\ell$. Then the original problem is reduced to finding a $\xi _\textsf {ref}(t)$ such that $\xi _\textsf {ref}(t) \downarrow p$ can avoid the bloated obstacles and reach the shrunk goal set.

### Use PWL Reference Trajectories for Vehicle Models

Finding a reference trajectory $\xi _\textsf {ref}(t)$ such that (a) $\xi _\textsf {ref}(t)$ satisfies the reach-avoid conditions, and (b) $\xi _\textsf {ref}(t)$ and $u_\textsf {ref}(t)$ are concatenations of state-input trajectory pairs $\{ (\xi _{\textsf {ref},i}, u_{\textsf {ref},i})\}_i$ and each pair satisfies the system dynamics, is a nontrivial problem. If we were to encode the problem directly as a satisfiability or an optimization problem, the solver would have to search for over the space of continuous functions constrained by the above requirements, including the nonlinear differential constraints imposed by *f*. The standard tactic is to fix a reasonable template for $\xi _\textsf {ref}(t), u_\textsf {ref}(t)$ and search for instantiations of this template.

From Example 4, we see that if $\xi _\textsf {ref}$ is a PWL reference trajectory constructed from waypoints in the workspace, the tracking error can be bounded using Lemma 2. A PWL reference trajectories connecting the waypoints in the workspace have the flexibility to satisfy the reach-avoid requirement. Therefore, in this section, we fix $\xi _\textsf {ref}$ and $u_\textsf {ref}$ to be the reference trajectory and reference input returned by the . In Sect. 5.2, we will see that the problem of finding such PWL $\xi _\textsf {ref}(t)$ can be reduced to a satisfiability problem over quantifier-free linear real arithmetic, which can be solved effectively by off-the-shelf SMT solvers (see Sect. 6 for empirical results).

### Synthesizing Waypoints for a Linear Reference Trajectory

Algorithm 1 says that $$\xi _\textsf {ref}(t)$$ and $$u_\textsf {ref}(t)$$ can be uniquely constructed given a sequence of waypoints $$\{p_i\}_{i=0}^k$$ in the workspace $$\mathcal{W}$$ and a constant velocity $$\bar{v}$$. From Proposition 1, $$p_\textsf {ref}(t) = \xi _\textsf {ref}(t) \downarrow p$$ connects the waypoints in $$\mathcal{W}$$. Also, let $$ t_i = \sum _{j=1}^{i} \Vert p_j-p_{j-1} \Vert _2/{\bar{v}}$$ be the concatenation time, $$\forall t \in [t_{i-1},t_{i})$$, *p*(*t*) is the line segment connecting $$p_{i-1} $$ and $$p_{i}$$. We want to ensure that $$p(t) = \xi _g(t)\downarrow p$$ satisfy the reach-avoid requirements. From Lemma 3, for any $$t \in [t_{i-1},t_{i})$$, we can bound $$\Vert p(t)-p_\textsf {ref}(t) \Vert _2$$ with the constant $$\ell _i$$, then the remaining problem is to ensure that, $$p_\textsf {ref}(t)$$ is at least $$\ell _i$$ away from the obstacles and $$p_\textsf {ref}(\xi _\textsf {ref}.\textsf {ltime})$$ is inside the goal set with $$\ell _k$$ distance to any surface of the goal set.

Let us start with one segment *p*(*t*) with $t \in [t_{i-1},t_{i})$. To enforce that *p*(*t*) is $\ell _i$ away from a polytope obstacle, a sufficient condition is to enforce both the endpoints of the line segment to lie out at least one surface of the polytope bloated by $\ell _i$.

#### Lemma 4

If $$p_\textsf {ref}(t)$$ with $$t \in [t_{i-1},t_{i})$$ is a line segment connecting $$p_{i-1} $$ and $$p_{i}$$ in $$\mathcal{W}$$. Given a polytope obstacle $$O = Poly(H_{{\scriptscriptstyle {O}}}, b_{{\scriptscriptstyle {O}}})$$ and $$\ell _i > 0$$, if

$$\begin{aligned} \bigvee _{s=1}^{\mathsf {dP}(H_{{\scriptscriptstyle {O}}})} \left( (H_{{\scriptscriptstyle {O}}}^{(s)} p_{i-1}> b_{{\scriptscriptstyle {O}}}^{(s)} + \Vert H_{{\scriptscriptstyle {O}}}^{(s)} \Vert _2\ell _i) \wedge ( H_{{\scriptscriptstyle {O}}}^{(s)} p_{i} > b_{{\scriptscriptstyle {O}}}^{(s)} + \Vert H_{{\scriptscriptstyle {O}}}^{(s)} \Vert _2\ell _i) \right) = \mathsf {True}, \end{aligned}$$

then $$\forall t \in [t_{i-1},t_{i})$$, $$B_{\ell _i}(p_\textsf {ref}(t)) \cap O = \emptyset $$.

#### Proof

Fix any *s* such that $(H_{{\scriptscriptstyle {O}}}^{(s)} p_{i-1}> b_{{\scriptscriptstyle {O}}}^{(s)} + \Vert H_{{\scriptscriptstyle {O}}}^{(s)} \Vert _2\ell _i) \wedge ( H_{{\scriptscriptstyle {O}}}^{(s)} p_{i} > b_{{\scriptscriptstyle {O}}}^{(s)} + \Vert H_{{\scriptscriptstyle {O}}}^{(s)} \Vert _2\ell _i)$ holds. The set $S = \{q \in {\mathbb R}^d \ | \ H_{{\scriptscriptstyle {O}}}^{(s)} q > b_{{\scriptscriptstyle {O}}}^{(s)} + \Vert H_{{\scriptscriptstyle {O}}}^{(s)} \Vert _2\ell _i\}$ defines a convex half space. Therefore, if $p_{i-1} \in S$ and $p_i \in S$, then any point on the line segment connecting $p_{i-1}$ and $p_{i}$ is in *S*. Therefore, for any $t \in [t_{i-1},t_{i})$, $H_{{\scriptscriptstyle {O}}}^{(s)} p_\textsf {ref}(t)> b_{{\scriptscriptstyle {O}}}^{(s)} + \Vert H_{{\scriptscriptstyle {O}}}^{(s)} \Vert _2\ell _i > b_{{\scriptscriptstyle {O}}}^{(s)}$, which means $p_\textsf {ref}(t) \notin O$.

The distance between $p_\textsf {ref}(t)$ and the surface $H_{{\scriptscriptstyle {O}}}^{(s)} q = b_{{\scriptscriptstyle {O}}}^{(s)}$ is $\frac{|H_{{\scriptscriptstyle {O}}}^{(s)} p_\textsf {ref}(t) - b_{{\scriptscriptstyle {O}}}^{(s)}|}{\Vert H_{{\scriptscriptstyle {O}}}^{(s)} \Vert _2} > \ell _i$. Therefore, for any $p \in B_{\ell _i}(p_\textsf {ref}(t))$ we have $\Vert p-p_\textsf {ref}(t) \Vert _2 \le \ell _i$ and thus $p \notin O$.

Furthermore, $\bigwedge _{s=1}^{\mathsf {dP}(H_{{\scriptscriptstyle {O}}})} H_{{\scriptscriptstyle {O}}}^{(s)} q \le b_{{\scriptscriptstyle {O}}}^{(s)} + \Vert H_{{\scriptscriptstyle {O}}}^{(s)} \Vert _2\ell _i$ defines of a new polytope that we get by bloating $Poly(H_{{\scriptscriptstyle {O}}}, b_{{\scriptscriptstyle {O}}})$ with $\ell _i$. Basically, it is constructed by moving each surface of $Poly(H_{{\scriptscriptstyle {O}}}, b_{{\scriptscriptstyle {O}}})$ along the surface’s normal vector with the direction pointing outside the polytope.

Similarly, we can define the condition when $p_\textsf {ref}(\xi .\textsf {ltime}) = p_k$ is inside the goal shrunk by $\ell _k$.

#### Lemma 5

Given a polytope goal set $G = Poly(H_{{\scriptscriptstyle {G}}}, b_{{\scriptscriptstyle {G}}})$ and $\ell _k > 0$, if

$$\begin{aligned} \bigwedge _{s=1}^{\mathsf {dP}(H_{{\scriptscriptstyle {G}}})} \left( H_{{\scriptscriptstyle {G}}}^{(s)} p_k \le b_{{\scriptscriptstyle {O}}}^{(s)} - \Vert H_{{\scriptscriptstyle {G}}}^{(s)} \Vert _2\ell _k \right) = \mathsf {True}, \ then \ B_{\ell _k}(p_k) \subseteq G. \end{aligned}$$

Putting them all together, we want to solve the following satisfiability problem to ensure that each line segment between $p_{i-1}$ and $p_{i}$ is at least $\ell _i$ away from all the obstacles and $p_k$ is inside the goal set *G* with at least distance $\ell _k$ to the surfaces of *G*. In this way, $\xi _g(t)$ starting from a neighborhood of $\xi _\textsf {ref}(0)$ can satisfy the reach-avoid requirement.Notice that the constraints in $\phi _\textsf {waypoints}$ are all linear over real arithmetic. Moreover, the number of constraints in $\phi _\textsf {waypoints}$ is $O\left( \sum \limits _{Poly(H,b) \in \mathbf{{O}}} k \mathsf {dP}(H) + \mathsf {dP}(H_{\scriptscriptstyle {G}}) \right)$. That is, fixing *k*, the number of constraints will grow linearly with the total number of surfaces in the obstacle and goal set polytopes. Fixing $\mathbf{{O}}$ and *G*, the number of constraints will grow linear with the number of line segments *k*.

#### Theorem 1

Fix $k \ge 1$ as the number of line segments, $p_\textsf {ref}(0) \in \mathcal{W}$ as the initial position of the reference trajectory. Assume that

1. $\mathcal{A}$ closed with $g_\textsf {ref}$ and $g_\textsf {trk}$ is such that given any sequence of $k+1$ waypoints in $\mathcal{W}$ and any $\bar{v}$, the piece-wise reference $\xi _\textsf {ref}$ (and input $u_\textsf {ref}$) returned by Algorithm 1 satisfy the conditions in Lemmas 2 and 3 with Lyapunov function *V*(*e*(*t*)) for the tracking error *e*(*t*).
2. For the above $\xi _\textsf {ref}$, fix an ${\varepsilon }_0$ such that $V(e(0)) \le {\varepsilon }_0$, let $\{ \ell _i \}_{i=1}^{k}$ be error bounds for positions constructed using Lemma 2 and Lemma 3 from ${\varepsilon }_0$.
3. $\phi _\textsf {waypoints}(p_\textsf {ref}(0),k,\mathbf{{O}},G,\{\ell _i\}_{i=1}^{k})$ is satisfiable with waypoints $\{p_i\}_{i=0}^{k}$.

Let  , and $p_\textsf {ref}(t) = \xi _\textsf {ref}(t) \downarrow p$. Let $\xi _g(t)$ be a trajectory of $\mathcal{A}$ closed with $g_\textsf {trk}(\cdot ,\xi _\textsf {ref},u_\textsf {ref})$ starting from $\xi _g(0)$ with $V(e(\xi _g(0),\xi _\textsf {ref}(0))) \le {\varepsilon }_0$, then $\xi _g(t)$ satisfies the reach-avoid requirement.

#### Proof

Since $\xi _\textsf {ref}(t),u_\textsf {ref}(t)$ are a PWL reference trajectory and a reference input respectively constructed from the waypoints $\{p_i\}_{i=0}^{k}$, they satisfy Assumption (1). Moreover, $V(e(\xi _g(0),\xi _\textsf {ref}(0))) \le {\varepsilon }_0$ satisfies Assumption (2). Using Lemma 2 and Lemma 3, we know that for $t \in [t_{i-1}, t_i), \Vert \xi _g(t)\downarrow p-\xi _\textsf {ref}(t)\downarrow p \Vert _2 \le \ell _i$.

Finally, since $\{p_i\}_{i=0}^{k}$ satisfy the constraints in $\phi _\textsf {waypoints}$, using Lemma 4 and Lemma 5, we know that for any time $t \in [0, t_k]$, $\xi _g(t)\downarrow p \notin \mathbf{{O}}$ and $\xi _g(t_k) \in G$. Therefore the theorem holds.

### Partitioning the Initial Set

Starting from the entire initial set $\Theta$, fix $\xi _\textsf {ref}(0) \in \Theta$ and an ${\varepsilon }_0$ such that $\forall x \in \Theta , V(e(x,\xi _\textsf {ref}(0))) \le {\varepsilon }_0$, then we can use Lemma 2 and Lemma 3 to construct the error bounds $\{ \ell _i \}_{i=1}^{k}$ for positions, and next use $\{ \ell _i \}_{i=1}^{k}$ to solve $\phi _\textsf {waypoints}$ and find the waypoints and construct the reference trajectory.

**Fig. 3.**
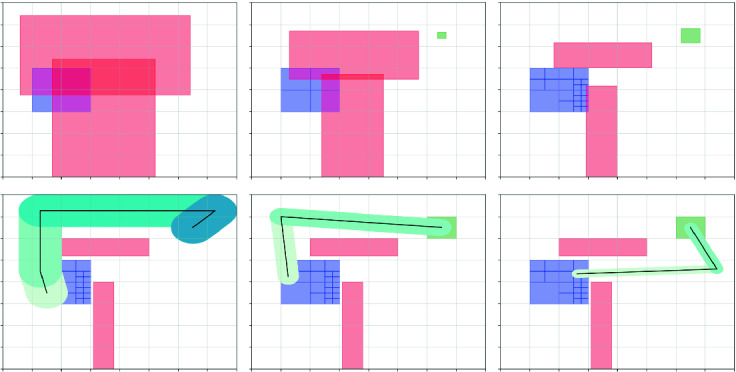
Top row: each step attempting to find a reference trajectory in the space where obstacles (goal set) are bloated (shrunk) by the error bounds $\{\ell _i\}_i$. From left to right: Without partition, $\{\ell _i\}_i$ are too large so a reference trajectory cannot be found. $\Theta$ is partitioned, but $\{\ell _i\}$s for the left-top cover are still too large. With further partions, a reference trajectory could be found. Bottom row: It is shown that the bloated tubes for each cover (which contain all other trajectories from that cover) can fit between the original obstacles.

However, if the initial set $\Theta$ is too large, $\{ \ell _i \}_{i=1}^{k}$ could be too conservative so $\phi _\textsf {waypoints}$ is not satisfiable. In the first two figures on the top row of Fig. 3, we could see that if we bloat the obstacle polytopes using the largest $\ell _i$, then no reference trajectory is feasible. In this case, we partition the initial set $\Theta$ to several smaller covers $\Theta _j$ and repeat the above steps from each smaller cover $\Theta _j$. In Lemma 2 and Lemma 3 we could see that the values of $\{ \ell _i \}_{i=1}^{k}$ decrease if ${\varepsilon }_0$ decreases. Therefore, with the partition of $\Theta$, we could possibly find a reference trajectory more and more easily. As shown in Fig. 3 bottom row, after several partitions, a reference trajectory for each $\Theta _j$ could be found.

### Overall Synthesis Algorithm

Taking partitioning into the overall algorithm, we have Algorithm 2 to solve the controller synthesis problem defined in Sect. 2.2. Algorithm 2 takes in as inputs (1) an (*n*, *m*)-dimensional control system $\mathcal{A}$, (2) a tracking controller $g_\textsf {trk}$, (3) Obstacles $\mathbf{{O}}$, (4) a goal set *G*, (5) a Lyapunov function *V*(*e*(*t*)) for the tracking error *e* that satisfies the conditions in Lemma 2 and Lemma 3 for any PWL reference trajectory and input, (6) the maximum number of line segments allowed $\textsf {Seg}_{\max }$, (7) the maximum number of partitions allowed $\textsf {Part}_{\max }$, and (8) a constant velocity $\bar{v}$. The algorithm returns a set $\mathtt {RefTrajs}$, such that for each triple $\langle \Theta _j, \xi _{j,\textsf {ref}}, u_{j,\textsf {ref}} \rangle \in \mathtt {RefTrajs}$, we have $\forall x_0 \in \Theta _j$, the unique trajectory $\xi _g$ of the closed system ($\mathcal{A}$ closed with $g_\textsf {trk}(\cdot ,\xi _{j,\textsf {ref}}, u_{j,\textsf {ref}})$) starting from $x_0$ satisfies the reach-avoid requirement. The algorithm also returns $\langle \mathtt {Cover}, \mathbf{None} \rangle$, which means that the algorithm fails to find controllers for the portion of the initial set in $\mathtt {Cover}$ within the maximum number of partitions $\textsf {Part}_{\max }$.
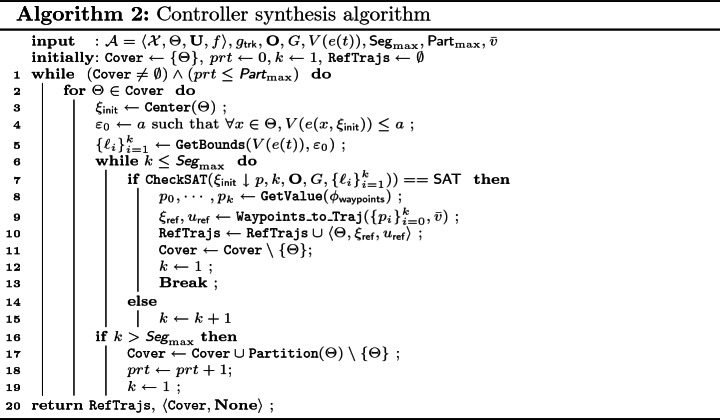


In Algorithm 2, $\mathtt {Cover}$ is the collection of covers in $\Theta$ that the corresponding $\xi _\textsf {ref}$ and $u_\textsf {ref}$ have not been discovered. Initially, $\mathtt {Cover}$ only contains $\Theta$. The **for**-loop from Line 2 will try to find a $\xi _\textsf {ref}$ and a $u_\textsf {ref}$ for each $\Theta \in \mathtt {Cover}$ until the maximum allowed number for partitions is reached. At line 3, we fix the initial state of $\xi _\textsf {ref}(0)=\xi _{\mathsf {init}}$ to be the center of the current cover $\Theta$. Then at Line 4, we get the initial error bounds ${\varepsilon }_0$ after fixing $\xi _{\mathsf {init}}$. Using ${\varepsilon }_0$ and the Lyapunov function *V*(*e*), we can construct the error bounds $\{\ell _i\}_{i=1}^{k}$ for the positions of the vehicle using Lemma 2 and Lemma 3 at Line 5.

If the **if** condition at Line 7 holds with $\{p_i\}_{i=0}^{k}$ being the waypoints that satisfy $\phi _\textsf {waypoints}$, then from Theorem 1 we know that the $\xi _\textsf {ref},u_\textsf {ref}$ constructed using $\{p_i\}_{i=0}^{k}$ at Line 9 will be such that, the unique trajectory $\xi _g$ of the closed system ($\mathcal{A}$ closed with $g_\textsf {trk}(\cdot ,\xi _{\textsf {ref}}, u_{\textsf {ref}})$) starting from $x_0 \in \Theta$ satisfies the reach-avoid requirement. Otherwise the algorithm will increase the number of segments *k* in the PWL reference trajectory (Line 15). When the maximum number of line segments allowed is reached but the algorithm still could not find $\xi _\textsf {ref},u_\textsf {ref}$ that can guarantee the satisfaction of reach-void requirement from the current cover $\Theta$, we will partition the current $\Theta$ at Line 17 and add those partitions to $\mathtt {Cover}$. At the same time, *k* will be reset to 1.

#### Theorem 2 (Soundness)

Suppose the inputs to Algorithm 2, $\mathcal{A}$, $g_\textsf {trk}$, $\mathbf{{O}}$, *G*, *V*(*e*(*t*)), $\bar{v}$ satisfy the conditions of Theorem 1. Let the output be $\mathtt {RefTrajs}= \{\langle \Theta _j, \xi _{j,\textsf {ref}}, u_{j,\textsf {ref}} \rangle \}_j$ and $\langle \mathtt {Cover}, \mathbf{None} \rangle$, then we have (1). $\Theta \subseteq \cup \Theta _j \cup \mathtt {Cover}$, and (2). for each triple $\langle \Theta _j, \xi _{j,\textsf {ref}}, u_{j,\textsf {ref}} \rangle$, we have $\forall x_0 \in \Theta _j$, the unique trajectory $\xi _g$ of the closed system ($\mathcal{A}$ closed with $g_\textsf {trk}(\cdot ,\xi _{j,\textsf {ref}}, u_{j,\textsf {ref}})$) starting from $x_0$ satisfies the reach-avoid requirement.

The theorem follows directly from the proof of Theorem 1.

## Implementation and Evaluation

We have implemented our synthesis algorithm (Algorithm 2) in a prototype open source tool we call FACTEST^5^ (FAst ConTrollEr SynThesis framework). Our implementation uses Pypoman^6^, Yices 2.2 [6], SciPy^7^ and NumPy^8^ libraries. The inputs to FACTEST are the same as the inputs in Algorithm 2. FACTEST terminates in two ways. Either it finds a reference trajectory $\xi _{j,\textsf {ref}}$ and reference input $u_{j,\textsf {ref}}$ for every partition $\Theta _j$ of $\Theta$ so that Theorem 2 guarantees they solved the controller synthesis problem. Otherwise, it terminates by failing to find reference trajectories for at least one subset of $\Theta$ after partitioning $\Theta$ up to the maximum specified depth.

### Benchmark Scenarios: Vehicle Models and Workspaces

**Fig. 4.**
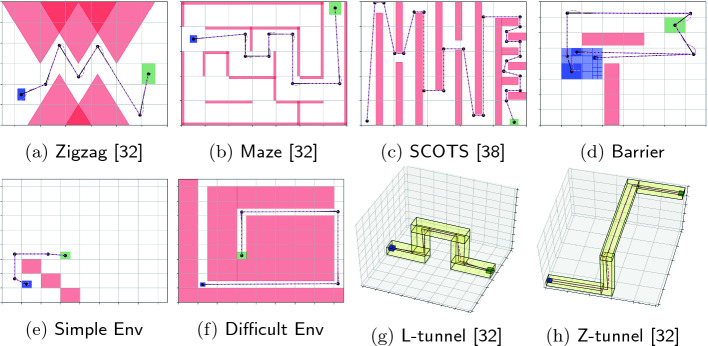
2D and 3D workspaces with initial (blue) and goal (green) sets. The scenarios run in the two-dimensional $\mathcal{W}$ use the car model. The scenarios run in the three dimensional $\mathcal{W}$ use the hovercraft model. The black lines denote $\xi _\textsf {ref}$ and the dotted violet lines denote $\xi _g$. (Color figure online)

We will report on evaluating FACTEST in several 2D and 3D scenarios drawn from motion planning literature (see Figs. 4). Recall, the state space $\mathcal{X}$ dimension corresponds to the vehicle model, and is separate from the dimensionality of the workspace $\mathcal{W}$. We will use four nonlinear vehicle models in these different scenarios: (a) the kinematic vehicle model (car) [31] introduced in Example 1, (b) a bijective mobile robot (robot) [13], (c) a hovering robot (hovercraft), and (d) an autonomous underwater vehicle (AUV) [29]. The dynamics and tracking controllers ($g_\textsf {trk}$) of the other three models are described on the FACTEST website [27]. Each of these controllers come with a Lyapunov function that meets the assumptions of Lemmas 2 and 3 so the tracking error bounds given by the lemmas $\{\ell \}_{i=1}^{k}$ can be computed.

### Synthesis Performance

Table 1 presents the performance of ${\textsf {FACTEST}}$ on several synthesis problems. Several points are worth highlighting. (a) The absolute running time is at the sub-second range, even for 6-dimensional vehicle models with 4-inputs, operating in a 3D workspace. This is encouraging for online motion-control applications with dynamic obstacles. (b) The running time is not too sensitive to dimensions of $\mathcal{X}$ and $\mathbf {U}$ because the waypoints are only being generated in the lower dimensional workspace $\mathcal{W}$. Additionally, the construction of $\xi _\textsf {ref}$ from the waypoints does not add significant time. However, since different models have different dynamics and Lypunov functions, they would have different error bounds for position. Such different bound could influence the final result. For example, the result for the Barrier scenario differs between the car and the robot. The car required 25 partitions to find a solution over all of $\Theta$ and the robot required 22. (c) Confirming what we have seen in Sect. 5.2, the runtime of the algorithm scales with the number of segments required to solve the scenario and the number of obstacles. (d) As expected and seen in Zigzag scenarios, all other things being the same, the running time and the number of partitions grow with larger initial set uncertainty.

**Table 1.** Synthesis performance on different scenarios (environment, vehicle). Dimension of state space $\mathcal{X}(n)$, input (m), radius of initial set $\Theta$, number of obstacles $\mathbf{{O}}$, running time (in seconds).

Scenario	n, m	Radius of $\Theta$	# $\mathbf{{O}}$	Time (s)	# segments per $\xi _\textsf {ref}$	# partitions
Zigzag, car 1	3, 2	0.200	9	0.037	6.0	1.0
Zigzag, car 2	3, 2	0.400	9	0.212	4.0	6.0
Zigzag, car 3	3, 2	0.800	9	0.915	5.0–6.0	16.0
Zigzag, robot 1	4, 2	0.200	9	0.038	6.0	1.0
Zigzag, robot 2	4, 2	0.400	9	0.227	4.0	6.0
Zigzag, robot 3	4, 2	0.800	9	0.911	5.0–6.0	16.0
Barrier car	3, 2	0.707	6	0.697	2.0–4.0	25.0
Barrier, robot	4, 2	0.707	6	0.645	2.0–4.0	22.0
Maze, car	3, 2	0.200	22	0.174	8.0	1.0
Maze, robot	4, 2	0.200	22	0.180	8.0	1.0
SCOTS, car	3, 2	0.070	19	1.541	26.0	1.0
SCOTS, robot	4, 2	0.070	19	1.623	26.0	1.0
L-tunnel, hovercraft	4, 3	0.173	10	0.060	5.0	1.0
L-tunnel, AUV	6, 4	1.732	10	0.063	5.0	1.0
Z-tunnel, hovercraft	4, 3	0.173	5	0.029	4.0	1.0
Z-tunnel, AUV	6, 4	1.732	10	0.029	4.0	1.0

**Comparison with Other Motion Controller Synthesis Tools: A Challenge.** Few controller synthesis tools for nonlinear models are available for direct comparisons. We had detailed discussions with the authors of FastTrack [11], but found it difficult to plug-in new vehicle models. RTD [44] is implemented in MatLab also for specific vehicle models. Pessoa [26] and SCOTS [38] are implemented as general purpose tools. However, they are based on construction of discrete abstractions, which requires several additional user inputs. Therefore, we were only able to compare FACTEST with SCOTS and Pessoa using the scenario SCOTS. This scenario was originally built in SCOTS and is using the same car model.

The results for SCOTS and Pessoa can be found in [38]. The total runtime of SCOTS consists of the abstraction time $t_{\text {abs}}$ and the synthesis time $t_{\text {syn}}$. The Pessoa tool has an abstraction time of $t_{\text {abs}} = 13509\,\text {s}$ and a synthesis time of $t_{\text {syn}} = 535\,\text {s}$, which gives a total time of $t_{\text {tot}} = 14044\,\text {s}$. The SCOTS tool has a has an abstraction time of $t_{\text {abs}} = 100\,\text {s}$ and a synthesis time of $t_{\text {syn}} = 413\,\text {s}$, which gives a total time of $t_{\text {tot}} = 513\,\text {s}$. FACTEST clearly outperforms both SCOTS and Pessoa with a total runtime of $t_{\text {tot}} = 1.541\,\text {s}$. This could be attributed to the fact that FACTEST does not have to perform any abstractions, but even by looking sole at $t_{\text {syn}}$, FACTEST is significantly faster. However, we do note that the inputs of FACTEST and SCOTS are different. For example, SCOTS needs a growth bound function $\beta$ for the dynamics but FACTEST requires Lyapunov functions for the tracking error.

### RRT vs. SAT-Plan

**Fig. 5.**
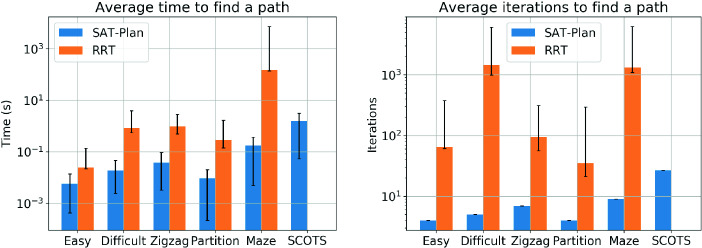
Comparison of RRT and SAT-Plan. The left plot shows the runtime and the right plot shows the number of necessary iterations. Note that RRT timed out on the SCOTS scenario.

To demonstrate the speed of our SAT-based reference trajectory synthesis algorithm (i.e. only the **while**-loop from Line 6 to Line 15 of Algorithm 2 which we call SAT-Plan), we compare it with Rapidly-exploring Random Trees (RRT) [20]. The running time, number of line segments, and number of iterations needed to find a path were compared. RRT was run using the Python Robotics library [39], which is not necessarily an optimized implementation. SAT-Plan was run using Yices 2.2. The scenarios are displayed in Fig. 4 and the results are in Fig. 5.

Each planner was run 100 times. The colored bars represent the average runtime and average number of iterations. The error bars represent the range of minimum and maximum. The RRT path planner was given a maximum of 5000 iterations and a path resolution of 0.01. SAT-Plan was given a maximum of 100 line segments to find a path. RRT timed out for the SCOTS scenario, unable to find a trajectory within 5000 iterations. The maze scenario timed out about 10% of the time.

Overall SAT-Plan scales in time much better as the size of the unsafe set increases. Additionally, the maximum number of iterations that RRT had to perform was far greater than the average number of line segments needed to find a safe path. This means that the maximum number of iterations that RRT must go through must be sufficiently large, or else a safe path will not be found even if one exists. SAT-Plan does not have randomness and therefore will find a reference trajectory (with *k* segments) in the modified space (bloated obstacles and shrunk goal) if one (with *k* segments) exists. Various examples of solutions found by RRT and SAT-Plan can be found on the FACTEST ’s website [27].

## Conclusion and Discussion

We introduced a technique for synthesizing correct-by-construction controllers for a nonlinear vehicle models, including ground, underwater, and aerial vehicles, for reach-avoid requirements. Our tool FACTEST implementing this technique shows very encouraging performance on various vehicle models in different 2D and 3D scenarios.

There are several directions for future investigations. (1) One could explore a broader class of reference trajectories to reduce the tracking error bounds. (2) It would also be useful to extend the technique so the synthesized controller can satisfy the actuation constraints automatically. (3) Currently we require user to provide the tracking controller $g_\textsf {trk}$ with the Lyapunov functions, it would be interesting to further automate this step.

## Footnotes

1 In the most recent International Conference on Robotics and Automation, among the 3,512 submissions “Path and motion planning” was the second most popular key phrase.

2 $\xi _g$ is a standard solution of ODE with piece-wise continuous right hand side.

3 A similar construction works for vehicles in 3D workspaces with additional variables.

4 $$\forall t \in [t_{i-1},t_{i}), \xi _\textsf {ref}(t) = \xi _{\textsf {ref},i}(t-\sum _{j=1}^{i-1}\xi _{\textsf {ref},j}.\textsf {ltime})$$.

5 All models and source code of FACTEST are available at [27].

6 https://pypi.org/project/pypoman/.

7 https://www.scipy.org/.

8 https://numpy.org/.
